# Gut Microbiota Is Not Significantly Altered by Radioiodine Therapy

**DOI:** 10.3390/nu17030395

**Published:** 2025-01-22

**Authors:** Pedro Barata, Ana Oliveira, Raquel Soares, Ana Fernandes

**Affiliations:** 1 RISE-Health, Faculdade de Ciências da Saúde, Universidade Fernando Pessoa, Fundação Ensino e Cultura Fernando Pessoa, Rua Carlos da Maia 296, 4200-150 Porto, Portugal; 2 Centro Hospitalar de Santo António, Unidade Local de Saúde de Santo António, Largo do Professor Abel Salazar, 4099-001 Porto, Portugal; 3 Department of Nuclear Medicine, Centro Hospitalar e Universitário de São João, E.P.E., 4200-319 Porto, Portugal; 4 Department of Biomedicine, Faculdade de Medicina da Universidade do Porto, 4200-319 Porto, Portugal

**Keywords:** shotgun metagenomics, radioiodine therapy, gut microbiota, ionizing radiation, thyroid cancer, hyperthyroidism

## Abstract

**Purpose:** Radiotherapy treatments are known to alter the gut microbiota. However, little is known regarding the effect of nuclear medicine treatments on gut microbiota, and it is established that nuclear medicine is inherently different from radiotherapy. To address this knowledge gap, we conducted a prospective study to identify changes in the gut microbiota of patients treated with [^131^I]NaI by comparing fecal samples before and after RAIT. **Methods:** Fecal samples of 64 patients (37 with thyroid cancer and 27 with hyperthyroidism) with indication for RAIT were collected 2 to 3 days before treatment and 8 to 10 days post-treatment. After DNA extraction, the gut microbiota’s richness, diversity, and composition were analyzed by shotgun metagenomics. In addition, LEfSe was performed to compare compositional changes in specific bacteria. **Results:** Gut microbiome richness and diversity remained unchanged after RAIT, with few changes in its composition identified, especially in patients with hyperthyroidism. **Conclusions:** This study provides a conceptual and analytical basis for increasing our understanding of the effects of radiopharmaceuticals on gut microbiota. Our preliminary results indicate that RAIT, contrary to radiotherapy, does not cause major disruptions to the human gut microbiota.

## 1. Introduction

The human gut microbiota comprises the microorganisms that collectively inhabit the intestinal tract [1,2]. Despite establishing its composition early in life and remaining relatively stable, it may suffer an imbalance—dysbiosis—precipitated by various factors [1,2,3]. It has been gaining attention from the scientific community for its role in digestion and, most importantly, for its association with (and possible causality of) multiple diseases [1], including inflammatory bowel disease, obesity, type 2 diabetes, cardiovascular diseases, and multiple cancers [1,2,3].

The gut microbiota has also been associated with the regulation of the response to treatments [4], including radiotherapy [2,5,6,7,8,9], and its composition has been used as a biomarker for outcome [10,11,12].

There is overwhelming evidence that gut microbiota is significantly altered by ionizing radiation [2]. Following exposure, possible tissue damage depends on the radiation dose and on the tissue’s radiosensitivity [2,13,14]. Indeed, some microorganisms present resistance to high levels of ionizing radiation [15,16].

Previous studies have demonstrated that ionizing radiation from contaminated areas and from abdominal and pelvic radiotherapy alter the gut microbiota of animals and humans [5,6,8,9,17,18,19,20].

Notwithstanding, nuclear medicine’s effects on the gut microbiota have not been explored and cannot be inferred from radiotherapy studies [18,21], since it is established that extrapolation from radiotherapy to nuclear medicine is not straightforward, because of differences in dose–rate effects, dosimetry, linear energy transfer, duration of treatment delivery, fractionation, range, and target volume [22,23,24].

Radioactive iodine therapy (RAIT) is a form of radiation therapy that consists of the systemic administration of radio-iodide ([^131^I]NaI) for selective irradiation of thyroid tissue. It is frequently used for the treatment of differentiated thyroid cancer (DTC) and of hyperthyroidism [25,26]. Ionizing radiation from [^131^I]NaI is highly energetic and penetrating and can alter the structure of molecules and atoms, affecting their function and eventually causing their destruction. [^131^I]NaI is administered orally and is mainly excreted by the kidneys and the colon [27]. RAIT is generally well-tolerated and safe but may be associated with side effects [28], including gastrointestinal ones (nausea, diarrhea, and vomiting) [25,29,30,31], indicating that some changes might occur in gut microbiota homeostasis. It follows that the gut microbiota plays an important role in [^131^I]NaI physiology and may represent an additional factor to response to RAIT therapy [32].

We previously investigated the effects of radiopharmaceuticals in an ex vivo approach and interestingly found an enrichment of important bacteria after RAIT [33,34,35]. However, the effect of RAIT on the gut diversity, richness, and composition of patients with hyperthyroidism and thyroid cancer is still unknown. To address this knowledge gap, we conducted a prospective study to identify changes in the gut microbiota of patients treated with [^131^I]NaI.

## 2. Materials and Methods

### 2.1. Study Population

A prospective study was conducted, enrolling 64 consecutive patients referred for RAIT from February 2019 to April 2021; 37, diagnosed with thyroid cancer, and 27 with hyperthyroidism.

Inclusion criteria were histologically confirmed differentiated thyroid carcinoma (upon total thyroidectomy) or biochemically confirmed hyperthyroidism; age ≥18 years.

Exclusion criteria were previous treatment with radioiodine or other nuclear medicine or radiation therapy; serious or unstable pre-existing medical condition that could interfere with compliance; treatments with antibiotics, steroids, immune suppressants, and pre- or probiotic in the previous six months.

All patients were treated according to the standards of clinical practice.

Prior to treatment, patients with thyroid cancer underwent four weeks of Levothyroxine withdrawal to stimulate TSH serum levels, except one, using Thyrogen^®^ (Cambridge, MA, USA); patients with hyperthyroidism underwent anti-thyroid medication withdrawal for five or more days.

Peripheral blood samples were collected just before treatment and at follow-up from thyroid cancer patients, and at follow-up from those with hyperthyroidism.

The activity of [^131^I]NaI ranged from 1110 MBq to 5550 MBq in patients with thyroid cancer, and from 222 MBq to 703 MBq in those with hyperthyroidism.

Demographic information, baseline characteristics and clinical parameters are summarized in Table 1.

Written informed consent was obtained from all patients. The study was performed in accordance with the Declaration of Helsinki and Good Clinical Practice guidelines following approval by the Centro Hospitalar Universitário de São João, E.P.E.’s and Faculdade de Medicina da Universidade do Porto ethics committees.

### 2.2. Sample Collection

Patients collected stool samples at two different time-points: 1 to 2 days before the treatment (pre-RAIT), and 8 to 10 days after the treatment (post-RAIT). In addition, patients completed a questionnaire at each time point to establish bowel patterns, concomitant medication, and dietary changes.

All samples were collected into a sterile plastic container and stored at the laboratory at −80 °C until further processing.

### 2.3. DNA Extraction and Sequencing

DNA from stool samples was isolated and quantified. DNA libraries were prepared using the Illumina Nextera XT library preparation kit, and library quantity was assessed with Qubit (Thermo Fisher Scientific™, Waltham, MA, USA). Libraries were then sequenced on an Illumina HiSeq platform (San Diego, CA, USA) 2 × 150 bp. 

### 2.4. Metagenomic Profiling and Statistical Analysis

Unassembled sequencing reads were directly analyzed using the CosmosID bioinformatics platform (CosmosID Inc., Rockville, MD, USA) for multi-kingdom microbiome analysis and quantification of an organism’s relative abundance

A high-performance data-mining k-mer algorithm was used. This algorithm disambiguates millions of short sequence reads into genomes that engender particular sequences. The pipeline includes two separable comparators: one consists of a pre-computation phase for reference databases, and the other is a per-sample computation.

The inputs to the pre-computation phase are databases of reference genomes that are continuously curated by CosmosID, and the output is a phylogeny tree of microbes combined with sets of biomarkers uniquely associated with distinct branches and leaves of the tree.

The second computational phase consists of searching the millions of short sequence reads, or, alternatively, contigs, from draft de novo assemblies against the biomarkers sets. This allows highly precise and sensitive detection and taxonomic classification of the NGS microbial reads. The obtained statistics are then analyzed to return the fine-grain taxonomic and relative abundance estimates for the microbial NGS datasets. These results are filtered using a threshold based on an internal statistical score, determined by analysis of multiple metagenomes, to exclude false positive identifications. The same approach is applied to enable the sensitive and accurate detection of genetic markers for virulence and resistance to antibiotics.

Assessment of significant differences alpha diversity was determined by the Wilcoxon rank-sum test. Alpha diversity was estimated using three indexes: Chao1 for microbial species richness and the Simpson and Shannon indexes for biodiversity and observed species. Additionally, permutational multivariate analysis of variance (PERMANOVA) was used for statistical significance testing of beta dissimilarities between selected groups. Beta diversity was assessed using Bray–Curtis distance-based non-metric multidimensional scaling analysis and the Jaccard dissimilarity index through 999 permutations. Diversity analyses were performed using species taxonomy level. A taxa bar plot was performed to distinguish discrepant abundant species between the different taxa of the groups, from the phylum to species level. To further explore key phylotypes that may contribute to the observed differences in microbial communities between cohorts, linear discriminant analysis (LDA) effect size (LEfSe) was performed to estimate differentially abundant features with biological consistency and statistical significance, with an LDA threshold value of >3.0. Statistical significance was considered for *p* < 0.05 values.

Statistical analysis was performed in R v4.1.2. The normality of the relative abundance difference distribution was tested using the Shapiro–Wilk test and by observing the histogram. Differences between relative bacterial abundance pre- and post-RAIT were tested using the Wilcoxon signals rank.

## 3. Results

### 3.1. Participants Characteristics

128 samples were analyzed from a total of 64 patients: 37 diagnosed with differentiated thyroid cancer and 27 with hyperthyroidism.

The thyroid cancer group includes 26 females and 11 males, ages 51 ± 17 years; the hyperthyroidism group includes 24 females and 3 males, ages 55 ± 15 years. The demographic and clinical characteristics of the two groups are presented in Table 1.

No significant differences were found when analyzing the gut microbiota composition at baseline concerning age, gender, BMI, or clinical indices parameters, including TSH (thyroid stimulating hormone), Tg (thyroglobulin), and TgAb (thyroglobulin antibody).

### 3.2. Impact of Radioiodine on the Gut Microbiota

In the study’s first phase, we analyzed if [^131^I]NaI affected the gut microbiota.

From the 128 fecal samples collected, 938,572,428 reads were generated after initial quality filtering, with an average of 7,332,597 reads per sample.

**Figure 1:**
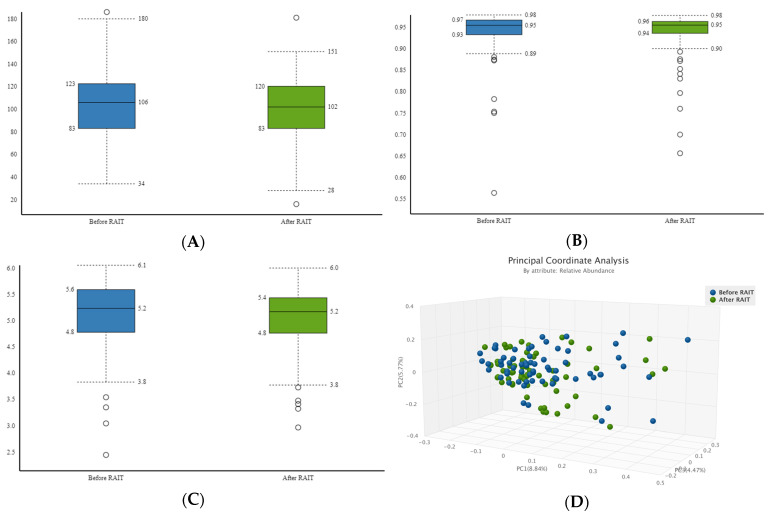

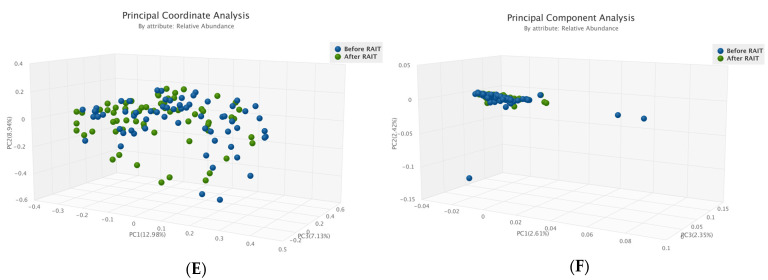
Analysis of diversity in samples pre-RAIT vs. post-RAIT. Alpha diversity was determined by (**A**) Chao1 index (*p* = 0.680); (**B**) Simpson index (*p* = 0.894) and (**C**) Shannon index (*p* = 0.454). Beta diversity was determined by PCoA analysis by (**D**) Jaccard distance (*p* = 0.362) and (**E**) Bray–Curtis (*p* = 0.050). 3D Principal Component Analysis (3D-PCA) plot (**F**). Each sphere represents a sample. Blue—pre-RAIT; Green—post-RAIT.

Alpha diversity, evaluated by Chao1, Simpson and Shannon indexes, was not significantly different between the pre- and post-treatment sets of samples (*p* = 0.680; *p* = 0.894; *p* = 0.454, respectively) (Figure 1A–C).

As shown in PCoA no separating trends between the bacterial communities in pre- and post-treatment groups were observed in terms of Jaccard and Bray–Curtis distances (*p* = 0.362 and 0.05, respectively) (Figure 1D,E). Similarity of microbiome community structures was further compared by a 3D-Principal component analysis (PCA) plot, which did not demonstrate differences in the visual coordinate data among the groups (Figure 1F).

After RAIT, we found a non-significant increase in the number of reads with a median of 10,919.71 reads and an IQR of −128,781.53 to 163,161.12 (*p* = 0.428).

Bacterial communities were explored at different taxonomic levels. A taxa bar plot showed the bacterial composition of each group’s phylum, family, genus, species and strains.

**Figure 2:**
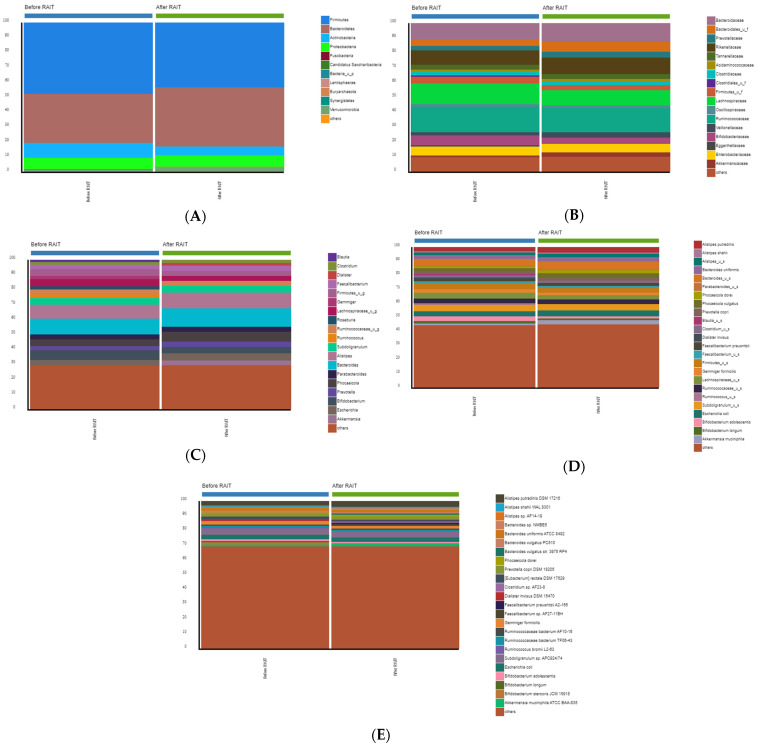
Box-plots of the composition of gut microbiota before and after RAIT samples. (**A**). The relative abundance of each phylum within each group/cohort is shown as a bar graph. The top 5 phyla were Firmicutes, Bacteroidetes, Proteobacteria, Actinobacteria and Verrucomicrobia. (**B**). Family level (others represent the 10% less abundant). (**C**). Genus level (“others” represent the 30% less abundant). (**D**). Species level (“others” represent the 45% less abundant). (**E**). Strain level (“others” represent the bottom 70% less abundant).

Five predominant phyla were identified: Actinobacteria, Bacteroidetes, Firmicutes, Proteobacteria, and Verrucomicrobia. However, Firmicutes and Bacteroidetes represented >80% in both pre- and post-treatment samples (Figure 2A). Firmicutes was the most abundant in both groups, making up an average of 47.85% and 43.52% of the total sequences of the gut microbiota of the samples before RAIT and after RAIT, respectively, followed by Bacteroidetes (32.99% and 39.47%, respectively) (Figure 2A). Nevertheless, it is noteworthy that microbial compositions showed high inter-individual variability. For example, Firmicutes accounted for 9.78–83.28%, Bacteroidetes 0.53–84.85%, and Actinobacteria 0.00–70.50% among all the individuals.

At phylum-level, we found a significant increase in Bacteroidetes after RAIT (median change 12.3%; IQR −16.7% to 129.6%; *p* = 0.010). No other significant differences were found in the relative abundances of the phyla between before and after treatment samples. In addition, we observed a decrease in the relative abundance of Actinobacteria and an increase in Verrucomicrobia, while Proteobacteria’s relative abundance was almost unchanged.

When analyzing the absolute abundance, significant increases were found for Proteobacteria (median change 31.2%; IQR −49.2% to 255.1%; *p* = 0.010) and Bacteroidetes (median change 41.6%; IQR −25.9% to 199.9%; *p* < 0.001).

At family level, the predominant taxa were *Bacteroidaceae* from the phylum Bacteroidetes, and *Lachnospiraceae* and *Ruminococcaceae* from the phylum Firmicutes (Figure 2B).

The relative abundance of each genus varied between individuals before and after RAIT samples (Figure 2C). The most abundant genera in the samples of pre-RAIT and post-RAIT were *Bacteroides* (10.76% and 12.56%, respectively) and *Alistipes* (9.19% and 10.57%, respectively). The third most frequent genus in the pre-RAIT samples was *Bifidobacterium* (6.66% and 4.26%), and on the contrary in post-RAIT samples was *Phocaeicola* (4.42% and 6.58%).

In analysis of the changes after RAIT, the data revealed that genus *Phocaeicola* was significantly overabundant in post-RAIT samples (median change 41.6%; IQR −29.6% to 217.9%; *p* = 0.021), possibly inflating the high relative abundance of Bacteroidetes at the phylum level, while *Lactobacillus*, from the Firmicutes phylum was more abundant in the pre-RAIT samples, decreasing in post-RAIT samples (median change −86.5%; IQR −100% to −26.2%; *p* = 0.021). No other significant changes were found in the relative abundance at genus level. However, when analyzing the absolute abundance, a significant increase was found in *Phocaeicola* (median change 33.7%; IQR −40.7% to 205.6%; *p* = 0.042) and in *Bacteroides* (median change 36.1%; IQR −26.5% to 208.6%; *p* = 0.042) and a significant decrease in *Lactobacillus* (median change −82.9%; IQR −100.0% to −13.7%; *p* = 0.021).

The relative abundance of the more abundant species is shown in Figure 2D. *Escheria coli* (3.56% and 4.38%), *Subdoligranulum_u_s* (4.60% and 4.40%), *Ruminococcaceae_u_s* (3.14% and 3.17%), *Firmicutes_u_s* (4.55% and 3.35%), and *Bacteroides_u_s* (4.28% and 5.49%) were the most frequent in the before and after RAIT samples. No significant findings were found in the relative and absolute abundances of the pre- and post-RAIT samples at species level.

LEfSe analysis revealed that the species *Alistipes inops*, *Ruminococcus bromii* and *Ruminococcus_u_s*, were significantly enriched in the fecal samples before treatment and were conversely less abundant after treatment. After treatment, *Bacteroides_u_s* and *Phocaeicola vulgatus* had notable increments in relative abundance. (Table 2).

### 3.3. Effect of RAIT in Patients with Hyperthyroism Versus Thyroid Cancer Patients

In the study’s second phase, we studied patients diagnosed with thyroid cancer and hyperthyroidism separately.

No significant differences were found in alpha diversity in the before and after treatment samples in both groups (Table 3).

**Figure 3:**
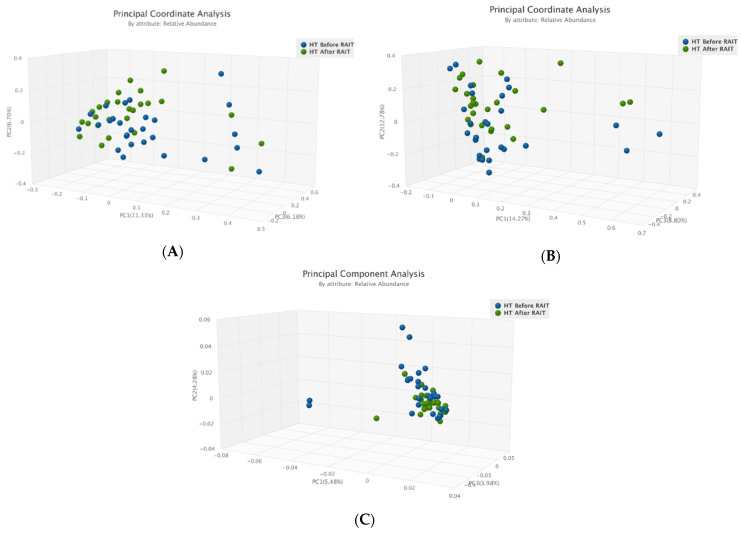
Beta diversity of the pre and post-samples of patients with hyperthyroidism. (**A**)—PCoA Jaccard. (**B**)—Bray-Curtis. (**C**)—3D-PCA.

**Figure 4:**
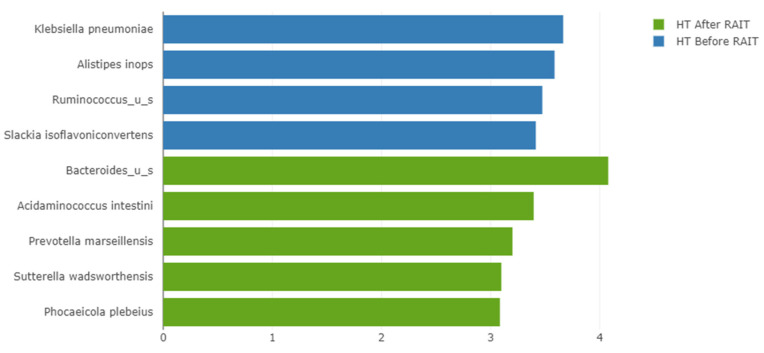
LEfSe bar-chart. Species enriched in patients with hyperthyroidism before (blue) and after (green) RAIT.

**Figure 5:**
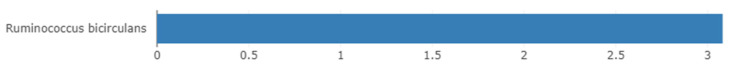
LEfSe bar-chart. In thyroid cancer patients, *Ruminococcus bicirculans* was enriched before treatment.

Differently, we found significant differences in Beta diversity in both Jaccard distance (*p* = 0.016) and Bray–Curtis dissimilarity (*p* = 0.030) between before and after RAIT samples of patients with hyperthyroidism (Figure 3, Figure 4 and Figure 5). Beta diversity did not show separation between the communities of each group of samples in thyroid cancer patients.

In the composition analysis, the only significant finding was the increase in the relative abundance in Bacteroidetes (IQR −11.8% to 439.1%; *p* = 0.010) in patients with hyperthyroidism. When analyzing the absolute abundance, we found increases of Proteobacteria with a median change of 128.3% (IQR −34.1% to 306.9%; *p* = 0.020) and of 117.1% for Bacteroidetes (IQR 6.1% to 1170.2%; *p* < 0.001). No significant changes were found in thyroid cancer patients at phylum-level.

At the genus level, no significant finding was found in the relative abundance of both groups. In the absolute abundance, we found a significant increase in Bacteroides, with a median percentual change of 95.2% (IQR −0.3% to 344.5%; *p* = 0.042) in patients with hyperthyroidism.

No significant changes were found at species-level for both studied groups.

In taxonomic analyses of patients with hyperthyroidism, LEfSe results revealed that Bacteroides_u_s, Acidaminococcus intestine, Prevotella marseillensis, Suterella wadsworthensis and Phocaeicola plebeius increased following RAIT and Klebsiella pneumonia, Alistipes inops, Ruminococcus_u_s and Slackia isaflavoniconvertens were significantly enriched before RAIT (Figure 4). In thyroid cancer patients LEfSe revealed that only Ruminococcus bicirculans was enriched before treatment (Figure 5).

## 4. Discussion

It’s known that radiotherapy markedly changes the gut microbiota, the most frequent finding being the loss of diversity and richness [36,37,38]. Systemic irradiation delivered via radiopharmaceuticals, however, is intrinsically different. As a consequence distinct radiation-induced biological responses are expected [22,24].

Previously we had found that ex vivo exposure to [^131^I]NaI causes the enrichment of relevant taxa in the human gut microbiota [33]. However, even though we used human feces, ex vivo setup considerably differs from the in vivo reality.

Therefore, we set to compare the gut microbiota from patients receiving RAIT to investigate its impact on the gut microbial community.

Our results revealed that differently from previous reports on the effect of ionizing radiation on the gut microbiota, the richness and diversity of fecal microbiota were not significantly different after radioiodine treatment.

We then compared the bacteria abundance shifts within the samples, and some significantly different bacteria taxa were identified.

As expected, the most represented phyla in the pre- and post-RAIT samples were Firmicutes and Bacteroidetes, followed by Proteobacteria, Actinobacteria, and Verrucomicrobia, the most commonly encountered bacterial phyla in the human intestinal tract. Their relative abundances differed between the pre and post-RAIT samples. The most relevant difference at phylum-level was a significant increase in Bacteroidetes causing a decrease in Firmicutes to Bacteroidetes (F/B) ratio (pre-RAIT 1.45 and post-RAIT 1.13). The F/B ratio shifts have been associated with several pathological conditions but more recent studies on lower taxonomic levels found this ratio not to be very reliable, arguing that the complexity of disease modulation by the gut microbiome must exceed the simple imbalance of two phyla. At lower taxonomic ranks, we found a significant decrease in *Lactobacillus* (*p* = 0.021), from the Firmicutes phylum. Additionally, through LEfSe analysis, we found that *Bacteroides_u_s* and *Phocaeicola vulgatus* were enriched after RAIT, and both belong to the Bacteroidetes phylum.

Regarding the less abundant phyla, we did not find significant statistical changes. Actinobacteria showed a decreasing trend post-RAIT, which can be explained by the reduction of the *Bifidobacterium* spp. (6.66% versus 4.42%) and the relative abundance of Verrucomicrobia exhibited an increasing tendency, which can be explained by the evident, but without statistical significance, increase in *Akkermansia muciniphila (A. muciniphila)*, from 1.21% to 2.99%. Two factors could explain this increase. First, this bacterium can tolerate small amounts of oxygen. Additionally, it belongs to the mucin-degrading bacterial family and can generate energy by producing enzymes that destroy mucin and use it as its sole carbon and nitrogen source. Due to these facts, when radiation induces increase in oxygen and mucin discharge, *A. muciniphila* has a competitive advantage over other bacteria [39,40].

Differently to previous studies, the relative abundance of Proteobacteria in our samples remained stable after irradiation (7.49% versus 7.48%). However, its absolute quantity increased significantly (*p* = 0.010). When intestinal inflammation occurs after IR exposure, oxygen levels are increased. This event leads to an increase in facultative aerobes such as Proteobacteria, which, unlike obligate anaerobic members of the gut microbiota, can use nitrate, S-oxides and N-oxides as terminal electron acceptors for anaerobic respiration [41,42,43].

Given that the overall profile of the gut bacterial community did not change significantly after radioiodine therapy, we then sought to identify differences between the effect of RAIT in patients with hyperthyroidism and thyroid cancer patients separately.

In thyroid cancer patients, we did not find significant differences in alpha and beta diversity and only *Ruminococcus bicirculans* was found to be enriched in the pre-RAIT samples. No other significant differences were found in composition.

In patients with hyperthyroidism, we found a significant difference between samples. Specifically, in the pre-RAIT samples four species were differentially enriched (*Klebsiella pneumoniae*, *Alistipes inops*, *Ruminococcus_u_s* and *Slackia isoflavoniconvertens*); in the post-RAIT samples, there were five (*Bacteroides_u_s*, *Acidaminococcus intestini*, *Prevotella marseillensis*, *Sutterella wadsworthensis*, and *Phocaeicola plebeius*). Additionally, we found significant increases in the relative and absolute abundances of Bacteroidetes, a significant increase in the absolute abundance of Proteobacteria, and a significant increase in the absolute abundance of *Bacteroides*. We concluded that the changes were due to hyperthyroidism.

Our limitations include the sample size, using fecal samples, which may not fully represent the structure of the mucosal microbiota, and this being a one-time-point microbiome study. We considered analysis in a time-series; however, the results would have a significant confounder, i.e., hypothyroidism or hyperthyroidism versus euthyroidism, and we could not have excluded that the hyperthyroid/hypothyroid/euthyroid state would does not influence the findings [44,45,46,47]. While the exclusion of patients taking antibiotics or probiotics aimed to reduce confounding, as well as the comparison of patients to themselves, we acknowledge that other factors, such as diet, lifestyle changes, and stress during the treatment period, were not systematically controlled, which may have influenced the observed outcomes and should be addressed in future studies.

## 5. Conclusions

Our study provides the first evidence that [^131^I]NaI does not significantly affect the richness and diversity of the human gut microbiota. This reinforces not only the safety of nuclear medicine treatments but also suggests that we should not extrapolate the radiobiology of external radiotherapy to nuclear medicine.

We found few significant shifts in the gut microbiota composition after RAIT only in the hyperthyroid patients’ group and concluded that most differences were attributable to variations in thyroid function.

## Figures and Tables

**Table 1:** Demographic and clinical characteristics (TMNG—toxic multinodular goiter; N/A—not applicable; ND—not determined).

Patients Characteristics	Thyroid Cancer Patients (*n* = 37)	Patients with Hyperthyroidism (*n* = 27)
Age (mean, range)	51 ± 17	55 ± 15
Gender		
Male	11	3
Female	26	24
BMI (mean, range)	28.3 ± 6.6	26.9 ± 7.2
Concomitant diseases		
Diabetes	7	4
Cardiovascular diseases	16	8
Other ^1^	8	6
Amount of chronic medication (mean, range)	3.1 ± 2.4	2.96 ± 2.58
Tumor Type		
Follicular	1	N/A
Papillar	36	N/A
Clinical T stage		
T1	16	N/A
T2	13	N/A
T3	6	N/A
T4	1	N/A
ND	1	N/A
Clinical N stage		
Nx	25	N/A
N0	4	N/A
N1	8	N/A
Clinical M stage		
M0	34	N/A
M1	3	NA
R		
R0	30	N/A
R1	5	N/A
R2	1	N/A
Cause of hyperthyroidism		
Grave’s disease	N/A	16
TMNG	N/A	6
Toxic adenoma	N/A	5
[^131^I]NaI dose		
185–370 MBq	N/A	8
371–555 MBq	N/A	17
556–703 MBq	N/A	2
1110 MBq	7	N/A
3700 MBq	22	N/A
5550 MBq	8	N/A

^1^ Psoriasis, chronic kidney disease, hyperuricemia, gastritis, etc.

**Table 2:** Taxonomic differences between the before and after RAIT samples analyzed through LEfSe analysis.

Species	Enriched Cohort	LDA Score	*p*-Value	Phylum
*Bacteroides_u_s*	After RAIT	3.708	0.02	Bacteroidetes
*Phocaeicola vulgatus*	After RAIT	3.697	0.036	Bacteroidetes
*Ruminococcus bromii*	Before RAIT	3.239	0.03	Firmicutes
*Ruminococcus_u_s*	Before RAIT	3.168	0.01	Firmicutes
*Alistipes inops*	Before RAIT	3.34	0.035	Bacteroidetes

**Table 3:** Alpha and Beta diversity *p* values of the before- and after-RAIT of patients with hyperthyroidism and thyroid cancer patients.

	Index	Hyperthyroidism	Thyroid Cancer
Alpha diversity	Chao1	*p* = 0.815	*p* = 0.456
	Simpson	*p* = 0.346	*p* = 0.273
	Shannon	*p* = 0.736	*p* = 0.208
Beta diversity	Bray-Curtis	*p* = 0.030	*p* = 0.994
	Jaccard	*p* = 0.016	*p* = 1.000

## Data Availability

The datasets generated during and/or analysed during the current study are available from the corresponding author on reasonable request. The data are not publicly available due to legal and ethical reason.
